# Insufficient Quality of Mental Health Information on German-Speaking TikTok: A Content Analysis

**DOI:** 10.32872/cpe.17279

**Published:** 2026-02-27

**Authors:** Aaron L. Mross, Hidehiko Takahashi, Katja Koelkebeck, Benedikt P. Langenbach

**Affiliations:** 1 LVR-University-Hospital Essen, Department of Psychiatry and Psychotherapy, Faculty of Medicine, University of Duisburg-Essen, Essen, Germany; 2 Department of Psychology and Psychotherapy, Witten/Herdecke University, Witten, Germany; 3 Center for Translational Neuro- and Behavioral Research (C-TNBS), Essen, Germany; 4 Department of Psychiatry and Behavioral Sciences, Graduate School of Medical and Dental Sciences, Tokyo Medical and Dental University, Tokyo, Japan; 5 Center for Brain Integration Research, Tokyo Medical and Dental University, Tokyo, Japan; 6 Bielefeld University, Medical School and University Medical Center OWL, Protestant Hospital of the Bethel Foundation, Department of Psychiatry and Psychotherapy, Bielefeld, Germany; Philipps-University of Marburg, Marburg, Germany

**Keywords:** TikTok, misinformation, ADHD, ASD, depression, anxiety, narcissism, PTSD

## Abstract

**Background:**

The increasing popularity of mental-health information on social media platforms such as TikTok is raising concerns regarding misinformation. Previous research is limited to single disorders and videos in the English language only. Our objective was to investigate the quality of mental health information on German-language TikTok for a broader spectrum of disorders.

**Method:**

Thirty German-language TikTok-videos of each of the six most viewed hashtags on mental disorders (attention-deficit and hyperactivity disorder (ADHD), depression, autism, anxiety disorder, narcissism and post-traumatic stress disorder (PTSD)) were classified regarding authorship and rated either as “correct”, “overgeneralized”, “incorrect” or “subjective experience”. The modified DISCERN (mDISCERN) and the Global Quality Scale (GQS) were used to rate reliability and quality of information for patients.

**Results:**

The 177 videos finally included in this study gathered a total of 94,348,220 views and 19.2% (*n* = 34) of the videos were rated as correct, 33.3% (*n* = 59) as incorrect, 18.1% (*n* = 32) as overgeneralized and 29.4% (*n* = 52) as personal experience. Chi-Square tests and Kruskal-Wallis tests showed significant relationships between either authorship or diagnosis and quality and reliability. Videos on PTSD and videos by expert authors showed the best and videos on narcissism and videos by laypeople the worst overall results.

**Conclusion:**

With around half of the analyzed videos supplying incorrect information, the quality of German-language TikTok mental health content is insufficient. Differences in the quality of content seem to be influenced by the topic and the authorship. Healthcare institutions and clinicians should be aware of this, educate patients accordingly, and could improve the quality of information by participating in online discourses.

## Highlights

- Videos on mental disorders are a viral topic on TikTok.
- Over 50% of the screened videos include incorrect or misleading information.
- Healthcare institutions, experts and therapists should educate patients accordingly.

“Narcissist do not love” or “people with ADHD love chaos” – statements like these fill countless videos on mental health topics on social media platforms such as TikTok. As one of the most downloaded and fastest growing social media platforms, TikTok is mostly used by younger people (ARD & ZDF, 2023; BBC News, 2021). Characterized by mainly self-produced, short video clips, TikTok is increasingly gaining public attention as a (dubious) source of information regarding (mental) health issues (Altrock & Kruckenhauser, 2022; Basch et al., 2022; Bonnen, 2024; Chochol et al., 2023; Clark, 2021; Haddadian, 2023; Kong et al., 2021; Lukić & Petrović, 2023; Sun et al., 2023).

The presentation of mental health on TikTok might lead to potential problems ranging from self-diagnosing to the glorification of mental disorders (Ahuja & Fichadia, 2024; Gilmore et al., 2022; Haltigan et al., 2023; Harness & Getzen, 2022; Monteith et al., 2024; Rettew, 2024). Even cases of a suspected social media-induced mass hysteria with tic-like symptoms have been reported (Giedinghagen, 2023; Müller-Vahl et al., 2022). Mental and public health information on TikTok yields potential advantages reaching young viewers but raises the question of whether viewers can distinguish between professional, correct information and misinformation (McCashin & Murphy, 2023). Further, the quality of information about mental disorders on TikTok is, itself, a cause for concern as studies on English language TikTok videos imply that the information in at least half of the videos on attention deficit and hyperactivity disorder (ADHD) and autism spectrum disorder (ASD) is misleading or just plain wrong (Aragon-Guevara et al., 2025; Karasavva et al., 2025; Yeung et al., 2022). Some studies even report around 90% of videos to be misleading (Verma & Sinha, 2025). Numbers that high can be a reason for concern. For other disorders, no systematic evaluation is available yet.

With mostly young viewers, these videos potentially reach an audience that has already been distressed by the COVID-19 pandemic (Blackwell et al., 2022; Jones et al., 2021; Panchal et al., 2023). This might be especially problematic for those who experience symptoms like attention deficits, anxiety, sleep problems or depressive mood, as they have already been shown to exhibit excessive social media use or a higher risk of social media misuse (Bickham et al., 2015; Bozzola et al., 2022; Hoge et al., 2017; Montag & Markett, 2024).

Thus, it is plausible to assume that this group might also turn to social media in an attempt to make sense of their symptoms but might then be faced with incorrect and misleading information, potentially leading to adverse effects. It is important to note that medical misinformation might lead to delayed or ineffective treatment, potentially prolonging and enhancing individual suffering and leading to chronification. As an example, a teenager who mistakes their symptoms of generalized anxiety disorder for ADHD symptoms might not seek appropriate therapeutic support. As Basch et al. (2022) argue, it is crucial to further examine prevalence and quality of mental health content on social media to understand possible effects on young people. Potential misassessment of disorders or symptoms by TikTok users could have important consequences for practitioners and their therapeutic interventions that need to be understood as well. For example, patients might adopt the incorrect use of professional jargon from social media (Chevalier, 2024), making mutual understanding between patient and practitioner more difficult.

To our knowledge, there has not yet been a study that has investigated the quality and possible differences of mental health information on TikTok for a broader spectrum of diagnoses, allowing a sufficient evaluation of content quality. However, as disorders differ in their social perception and theoretical complexity, it is likely that videos concerning these disorders also differ in accuracy. Compared to the complex scientific theories on ADHD, the origin of a post-traumatic stress disorder (PTSD), might be much easier to explain in a short video as, for example, a result of something specific that happened to someone. With only English videos having been analyzed to date, the situation for other languages and cultural areas remains unknown. Thus, our aim for this study was to examine the quality of information on the most popular mental health topics on German-speaking TikTok. In contrast to previous research, we expanded the analysis to a broader number of different disorders. Similar to previous studies, we expected a large proportion of videos with incorrect content. In addition, we expected differences in the quality of information between different disorders. We also investigated whether authorship might influence the quality of content (e.g., videos by mental health experts being more correct than those made by laypeople).

## Method

### Sample

**Figure 1:**
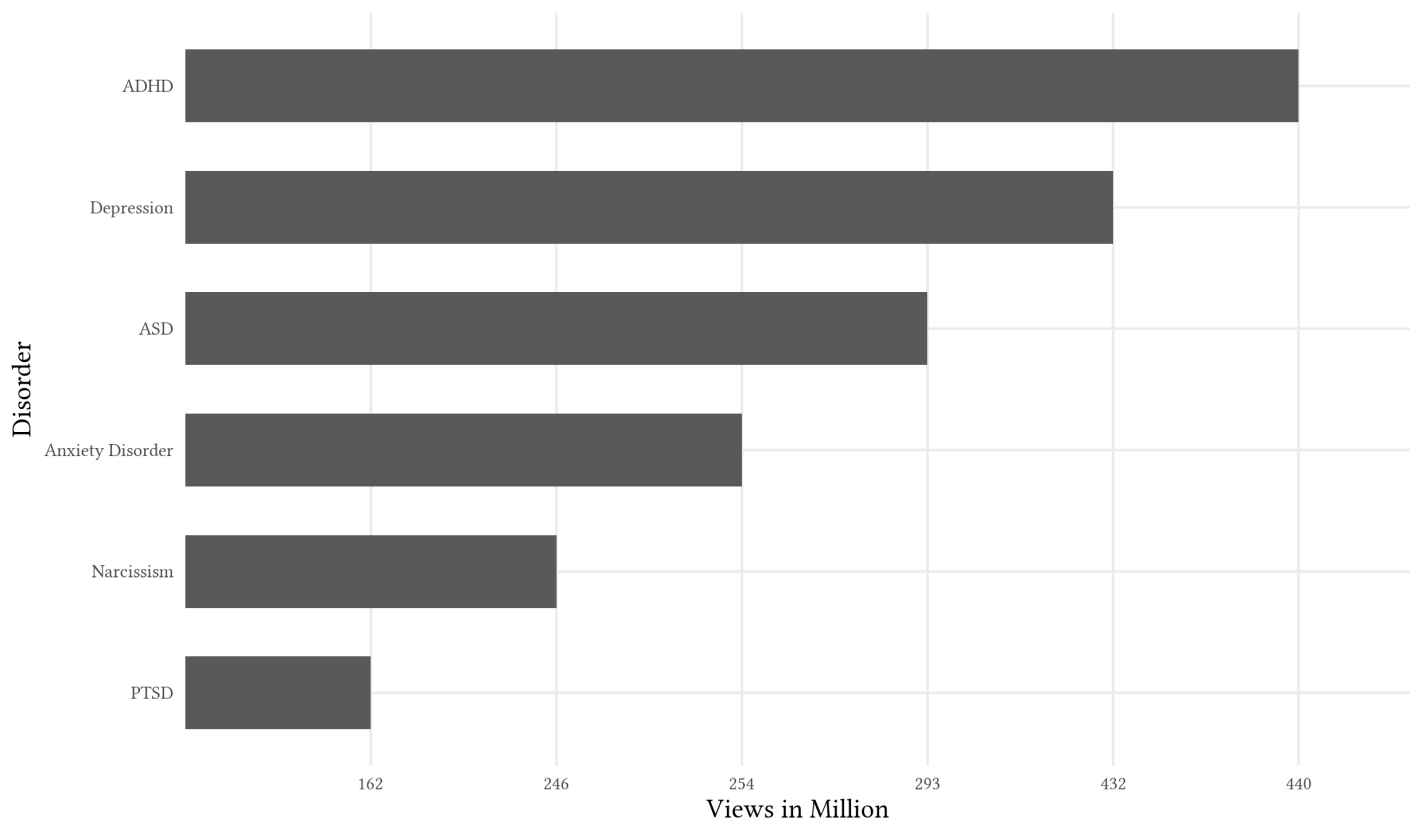
Overview of German-Language Hashtags Related to Mental Health on TikTok With the Highest Cumulative View Counts *Note.* The six most viewed hashtags on mental disorders on German-language TikTok on January 4th, 2024. Original German hashtags were: #ADHS; #Depression; #Autismus; #Angststörung; #Narzissmus; #PTBS.

To achieve a comprehensive overview of viral mental health topics on TikTok, a TikTok account was created to follow the six German-language hashtags about mental disorders that have the highest overall views (see Figure 1).

For each disorder, the first 30 German videos were included, sorted by the proprietary TikTok app algorithm. Exclusion criteria were languages other than German, videos unrelated to the disorder, and videos not including information about the disorder that could, accordingly, not be evaluated by the rating criteria used in this study. From January 4th, 2024, to February 14th, 2024, a total of 180 videos were downloaded for assessment via a free online tool (https://ssstik.io/). Three videos were excluded, resulting in 177 included videos. Videos were excluded as not ratable with reference to diagnostic criteria for the following reasons: one video was only comprised of motivational speech such as “you are valuable”, one gave advice on how to behave towards depressed individuals, and one gave information about genetic predisposition of ASD. No intended sample size was calculated but we conducted a sensitivity analysis in G*Power (Faul et al., 2007) revealing that the current sample was sufficiently powered (1-β = .80, α = .05) to detect small to medium effects (w = .277). Meta data was extracted as visible on the TikTok app, supplying rounded-up numbers for views, likes and comments. The procedure was chosen to be representative of the experience a user might have searching for a diagnosis on TikTok.

### Rating Procedure

Ratings to assess the quality of the content were independently conducted by two clinical psychologists with diagnosis experience in both in- and outpatient settings (AM, BL). In keeping with previous, comparable research (Aragon-Guevara et al., 2025; Yeung et al., 2022), videos were rated as (a) correct, (b) overgeneralized, (c) incorrect or describing (d) subjective experience, to allow comparability of results. Videos that contained both incorrect information as well as subjective descriptions were rated as incorrect. Authorship of the video was rated as (a) expert, (b) affected individual or (c) layperson, according to the information given in the videos. For detailed information on the categories, see Table 1.

**Table 1:** Explanation of the Rating Levels Used in This Study

Category	Explanation
Correct	Presents core symptoms of and information on the disorder correctly
Overgeneralized	Presents symptoms and information that are typical for mental disorders but do not belong to core symptoms of the disorder or describes undifferentiated and overgeneralized symptoms
Incorrect	Presents symptoms that are not symptoms of the disorder or common phenomena of daily life or wrong information
Subjective experience	Subjective and personal experience by affected individuals
Expert	An expert who (by self-declaration) underwent an officially regulated education or attended an official healthcare institution regulated by German social law (e.g. psychologist or psychotherapist, medical doctor, psychiatric nurse)
Affected person	A person who received the diagnosis by an official healthcare institution/expert
Layperson	A person who is neither of the above. This includes alternative practitioners and coaches, who by German law do not have to undergo professional and regulated education or training

Differences in the ratings were later discussed by the two mentioned raters, and a final rating was conducted by mutual consent. The Kappa statistic of the initial quality ratings was .813 (*p* < .001), indicating very good inter-rater reliability. A decision by mutual consent was required for 19 videos, 17 of which differed by just one rating level (e.g. misinformation – overgeneralized).

### Standardized Assessments

For a quantitative assessment of the quality of the information for patients, videos were rated by the same two raters on the Global Quality Scale (GQS; Steeb et al., 2022; Uzun, 2023), a five-point Likert scale ranging from “very poor quality” to “excellent quality” based on video quality, flow of information (e.g. comprehensible sequence of information, no abrupt changes in topic) and usefulness of information. Reliability of the information provided in the videos was assessed using the modified DISCERN (mDISCERN), a shortened version of the DISCERN (Charnock et al., 1999; Singh et al., 2012; Uzun, 2023), supplying a five-point Likert scale ranging from “unreliable” to “very reliable”. The mDISCERN consists of five yes/no-questions, leading to a maximum of five points on the scale (with higher values corresponding to better quality). Questions referred to understandability, validity of cited sources, unbiased/balanced content, additional sources, and mention of uncertainty (for both GQS and mDISCERN, see the Supplementary Materials). Both scales were chosen to allow future research to achieve comparability and are already frequently used in similar research on medical information. Furthermore, we think that a more viewer-focused scale (GQS) combined with a more science-focused measure (mDISCERN) covers two important aspects of mental-health information on social media.

### Statistic Procedure

Statistical analyses were performed in SPSS v29. For the inter-rater reliability, Cohen’s Kappa was calculated on the initial independent ratings. To examine if the quality of the content was associated with authorship and disorder, non-parametric chi-square contingency analyses with Cramér’s V were calculated as a measure for strength of the association. Chi-square tests allow a calculation of an association between two categorial variables via a comparison of the found distribution with the theoretically expected one. Kruskal-Wallis-Tests with Bonferroni-corrected significance levels were used to examine the associations with the GQS and mDISCERN, assuming non-normally distributed data. Effect sizes for the Kruskal-Wallis-Tests were reported according to Cohen (2009). Kruskal-Wallis is a nonparametric test that can be used to calculate differences between two or more groups when the data is not normally distributed and, thus, criteria for an ANOVA are not met.

### Ethical Consideration

With only publicly available material being included and consistent with previous comparable research (Yeung et al., 2022; Zea Vera et al., 2022) as well as in accordance with the German law, no ethics approval was obtained. No identifiable information or personal data is included in this manuscript.

## Results

### Basic Video Characteristics

The 177 rated videos had a total amount of 94,348,220 views with an average of 533,040 (range 569 – 15,300,000) per video and 7,401,638 likes in total with an average of 41,817 (range 116 – 1,100,000) per video. Thirty-two (18.1%) of the videos were uploaded by experts, 88 (49.7%) by affected persons and 57 (32.2%) by laypeople. Of the 177 Videos, 19.2% (*n* = 34) were rated as correct, 33.3% (*n* = 59) as incorrect, 18.1% (*n* = 32) as overgeneralized and 29.4% (*n* = 52) as personal experience. The videos showed an average GQS of 1.82 (*SD* = .966, range 1 – 5) and mDISCERN of 1.34 (*SD* = .655, range 1 – 4). The detailed video characteristics by authorship and diagnosis are listed in Table 2. No statistically significant correlations between views and likes for authorship, rating or diagnosis were found.

**Table 2:** Detailed Video Characteristics by Diagnosis and Authorship

Variable	View		Likes		GQS		mDISCERN	
	*M*	*SD*	*M*	*SD*	*M*	*SD*	*M*	*SD*
Expert	321,434.59	520,054.03	29,438.06	616,607.76	2.37	1.04	1.66	.75
Affected	517,502.63	1,691,093.59	43,950.55	128,422.65	1.94	1	1.35	.64
Layperson	675,826.00	977,509.45	45,473.19	97,989.21	1.33	.58	1.15	.56
ADHD	1,037,291.30	2,781,738.19	82,927.27	209,378.81	1.60	.76	1.24	.52
Anxiety	781,654.45	1,065,755.75	81,498.52	127,779.59	1.52	.75	1	0
ASD	395,728.90	584,023.51	23,520.28	30,548.51	1.81	.88	1.37	.74
Depression	236,367.90	481,821.85	27,457.86	64,463.19	1.90	.98	1.52	.83
Narcissism	634,197.23	857,910.16	26,257.67	45,173.47	1.17	.38	1	0
PTSD	106,825.93	222,304.63	9,475.57	23,038.96	2.93	1.03	1.93	.70

*Note.* PTSD = post-traumatic stress disorder; ADHD = Attention-Deficit and Hyperactivity Disorder; ASD = Autism-Spectrum-Disorder; Anxiety = includes anxiety disorders such as social anxiety, panic disorder and generalized anxiety disorder from the DSM-5; Depression = includes all depressive disorders from the DSM-5.

### Association of Quality of Content and Authorship

**Figure 2:**
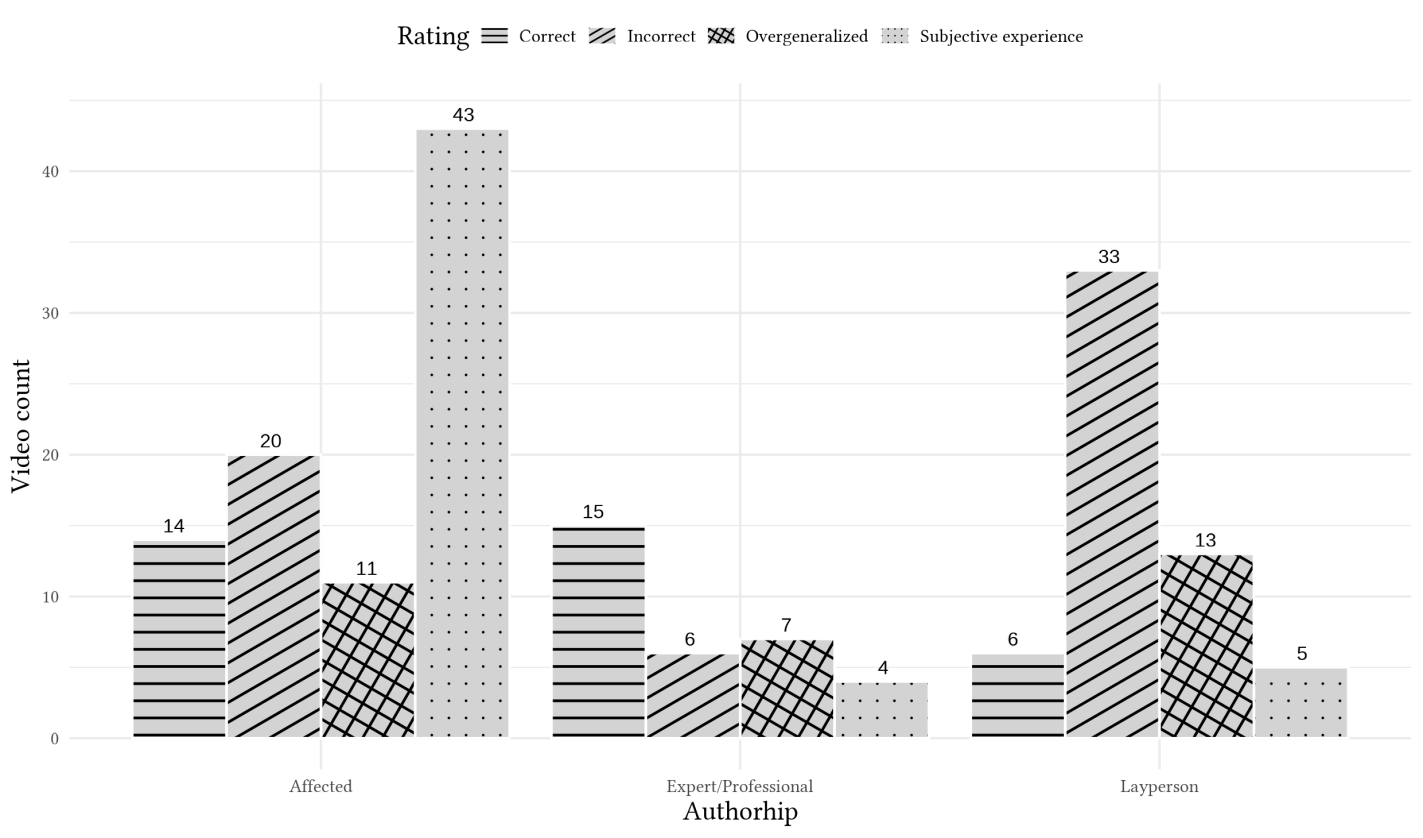
Ratings of the Videos by Authorship, Count in Number of Videos

The differences in quality of content based on authorship can be seen in Figure 2. A Chi-square test of independence revealed that the rated quality of information differed statistically significantly by authorship (χ^2^(6, *n* = 177) = 57.618, *p* = .001), showing medium associations (*CC* = .496, *p* < .001; Cramér’s V = .403, *p* < .001). The GQS differed significantly dependent on authorship (H(2) = 26.789; *p* < .001). Post-hoc Dunn-Bonferroni-Tests showed statistically significant differences between Layperson and Expert (*z* = 4.913, *p* < .001) with a medium effect *r* = .378 as well as between Layperson and Affected (*z* = 3.743, *p* = .001) with a small effect *r* = .288. Differences between Expert and Affected remained insignificant for the GQS. The mDISCERN differed statistically significantly depending on authorship as well (H(2) = 18.419; *p* < .001). Post-hoc Dunn-Bonferroni-Tests showed significant differences between all groups (Layperson and Expert (*z* = 4.261, *p* < .001), *r* = .328; Layperson and Affected (*z* = 2.510, *p* = .036), *r* = .193; Affected and Expert (*z* = 2.446, *p* = .043), *r* = .188) showing only small effects except for Layperson and Expert.

### Association of Quality of Content and Disorder

**Figure 3:**
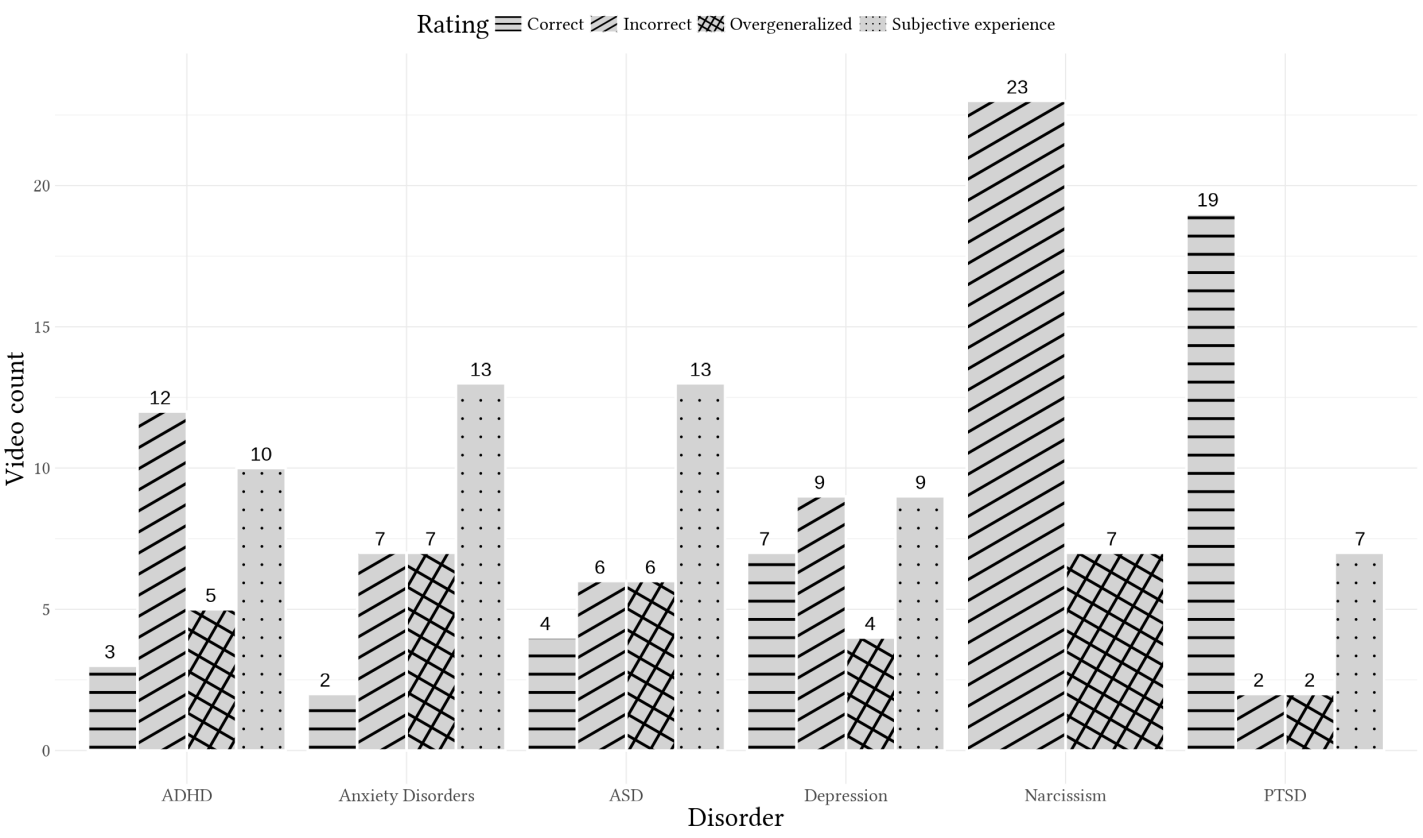
Ratings of the Videos by Disorder, Count in Numbers of Videos *Note.* ADHD = Attention-Deficit and Hyperactivity Disorder; ASD = Autism-Spectrum-Disorder; PTSD = post-traumatic stress disorder.

The differences in quality of content for different disorders can be seen in Figure 3. A Chi-square test of independence revealed that the rated quality of content differed statistically significantly by diagnosis (χ^2^(15, *n* = 177) = 78.679, *p* = .001), showing a medium association (*CC* = .555, *p* < .001; Cramér’s V = .385, *p* < .001). The GQS differed significantly dependent on diagnosis (H(5) = 48.795; *p* < .001). Post-hoc Dunn-Bonferroni-Tests showed significant differences between Narcissism and Depression (*z* = 3.107, *p* = .028) with a small effect *r* = .239, Narcissism and PTSD (*z* = -6.699, *p* < .001) with a strong effect *r* = .515, Anxiety Disorders and PTSD (*z* = -4.809, *p* < .001) with a medium effect *r* = .370, ADHD and PTSD (*z* = -4.443, *p* < .001) with a medium effect *r* = .342, ASD and PTSD (*z* = -3.694, *p* = .003) with a small effect *r* = .284 and between Depression and PTSD (*z* = -3.562, *p* = .006) with a small effect *r* = .274. The mDISCERN differed statistically significantly dependent on diagnosis (H(5) = 48.795; *p* <. 001). Post-hoc Dunn-Bonferroni-Tests showed significant differences between Narcissism and Depression (*z* = 3.087, *p* = .030) with a small effect *r* = .237, Narcissism and PTSD (*z* = -6.259, *p* < .001) with a medium effect *r* = .481, Anxiety Disorders and Depression (*z* = -3.061, *p* = .033) with a small effect *r* = .235, Anxiety Disorders and PTSD (*z* = -6.207, *p* < .001) with a medium effect *r* = .477, ADHD and PTSD (*z* = -4.357, *p* < .001) with a medium effect *r* = .335, ASD and PTSD (*z* = -3.889, *p* = .002) with a small effect *r* = .299 and between Depression and PTSD (*z* = -3.146, *p* = .025) with a small effect *r* = .242.

## Discussion

In this study, a total of 177 TikTok videos about mental disorders were analyzed regarding content quality and reliability. This study was the first to cover videos on six different disorders, gathering a total of almost 100,000,000 views. Our results show that more than half of the videos were incorrect or overgeneralized, raising concerns about the general quality of mental health information on social media. The ratings showed differences depending on authorship, with layperson being more strongly associated with incorrect videos, lower quality for patients and less reliability.

The percentage of incorrect videos also varied between different disorders, with videos on PTSD being substantially more correct than those on any other disorder investigated. Then again, every single video on narcissism contained misinformation. This study is the first to report differences in quality and reliability between videos on different disorders on social media. Furthermore, it is the first study showing the insufficient quality of information on mental disorders on TikTok in a non-English-speaking sample. The results are in line with previous research on mental health TikTok videos (Aragon-Guevara et al., 2025; Karasavva et al., 2025; Verma & Sinha, 2025; Yeung et al., 2022) and other medical topics (Chochol et al., 2023; Kong et al., 2021; Lukić & Petrović, 2023; Sun et al., 2023) in English.

Our results also show that mental illness is a viral topic on social media, largely driven by affected persons themselves: they not only produce most of the videos rated as subjective experience, but also just short of half of all the videos in this study. It seems as if social media platforms such as TikTok are vastly used by affected individuals to share their knowledge and experiences. Indeed, patients might benefit from a virtual space to share experiences and offer mutual support. On the other hand, this creates a new social sphere where (mis)information about mental health topics can circulate without any professional or scientific curation. Without guidance, it is hard to differentiate between valid, useful information and incorrect, possibly harmful information (see Table 3). This could be further complicated when some disorders are predominantly presented using correct information, while others are not. In our sample, videos on PTSD showed significantly better ratings of quality compared to all other disorders, also registering the most experts as authors. On the other hand, videos on narcissism were almost exclusively produced by laypeople, showing exclusively incorrect or overgeneralized information.

**Table 3:** Example Sentences From Videos Including Misinformation

ADHD	“Trigger points for people with ADHD: tangled cables, slow technical devices, things that don’t work immediately, getting told what to do.”
	“ADHD is not a curse – it is a superpower.”
Anxiety	“Symptoms of an anxiety disorder that most people don’t understand: eating too much or too little … “
ASD	“Things I do that are autistic: I have no sense of orientation and struggle to find my way around new places … “
Depression	“Men with depression” – video showing famous male movie characters smiling
	“It is extremely difficult [for a depressed person] to feel and show love.”
Narcissism	“Narcissist do not love.”, “They only want to use [people]”
	“Narcissists have a very limited vocabulary.”

*Note*. ADHD = Attention-Deficit and Hyperactivity Disorder; ASD = Autism-Spectrum-Disorder.

It can be assumed that affected people with stigmatized disorders are less likely to present themselves and supply information publicly. These differences in authorship seem to be resulting in a skewed representation of information for certain disorders. For example, PTSD can be seen as a hardship that happens to a person or is done to them. Thus, a person with PTSD might (quite correctly) portray themselves as the victim of objectively negative circumstances. On the other hand, the videos in our sample often insinuated that those affected by a narcissistic personality disorder are lacking good morals and ethics and are incapable of love and positive emotions (in stark contrast to the fact that individuals with a narcissistic personality disorder have often been victims of abuse themselves (Bertele et al., 2022; Clemens et al., 2022; Gao et al., 2024)). Identifying with the latter disorder online might thus be less appealing and results, in general, in fewer videos produced by people affected by stigmatized disorders.

Another potential explanation why videos on PTSD are more reliable could be the nature of its symptoms. Experiencing a traumatic event and having flashbacks is a relatively easy-to-grasp and clearly defined symptom. With other disorders, symptoms might sometimes be more vaguely defined (e.g., “deficits in reciprocal social communication and social interaction” (APA, 2013) in ASD) or seem to resemble everyday phenomena more strongly: many people will know what it is like to be easily distracted, even if they do not have a diagnosis of ADHD.

It can only be speculated why such a large amount of information on TikTok is relatively untrustworthy. One potential reason could be the misunderstanding of scientific facts. For example, “object permanence” was falsely interpreted as an ADHD symptom to explain difficulties with remembering objects or appointments. It turned into a viral talking point on social media (Chevalier, 2024) and was widely presented as a symptom of ADHD, when it is actually describing the ability of a child to understand that objects or persons continue to exist even if they cannot be seen or perceived (Barrett, 2001). Similarly, someone could self-diagnose with a disorder after referring to information online and then present their own, maybe unrelated symptoms as part of that disorder. When such information is then spread, cited and repetitively linked to a disorder, new concepts of psychiatric terms and disorders could emerge without science being involved, at all.

The effects on patients and professionals remain unclear. The most obvious consequences could be patients in need of therapy not seeking help after being convinced that, e.g., “ADHD is a superpower”. Others could seek (and possibly receive) therapy of an incorrectly self-diagnosed disorder. With over 90% of TikTok videos referring to tests on ADHD consisting of misleading and incorrect tests and information (Verma & Sinha, 2025), viewers might easily be misled into self-diagnosing a serious mental disorder. If an individual has already received a diagnosis and is then presented with highly negative, conflicting content online, this could have adverse effects on their self-image as well. One can imagine how someone suffering from a narcissistic personality disorder would feel about themself, being characterized as “unable to love” and wanting “to use people”. Further, disorders that are presented as more favorable, popular, or less severe in certain online communities could put additional stress on affected individuals when they do not fit into popular narratives on social media or suffer from a disorder that is presented as less acceptable. Someone suffering from obsessive-compulsive disorder might be presented with information about routines or repetitive behavior in ASD with a focus on self-acceptance and suddenly feel conflicted about their own diagnoses or symptoms. As a result, there could be a (possibly unintentional) attempt to fit into a certain social-media narrative, despite it not representing real-life experience.

Further, as the design of social media platforms increases the risk of problematic social media use, linked to traits such as neuroticism and impulsivity (Sindermann et al., 2022). In some cases, there might be a vicious circle between social media use and psychopathological symptoms. The possible extent and impact of this can be observed in the cases of functional tics and tic-like behaviors (Giedinghagen, 2023; Müller-Vahl et al., 2022; Olvera et al., 2021). All these examples could lead to unnecessarily prolonged suffering or even worsening of symptoms over time as a consequence of incorrect social-media information on mental health.

### Conclusion

Overall, this study supplies a detailed overview of the quality and reliability of information on mental disorders on (German-language) TikTok, indicating important differences between authors and disorders. Research needs to focus more on why such large amounts of misinformation are viral and how this affects potential viewers. Practitioners need to be made aware of the content available online and be open to talk about misinformation with their patients, also providing help in how to identify “correct” content. As only one of the videos we rated was produced by an official institution, healthcare institutions could take the initiative and supply professional information on mental health topics on social media to enhance the chances for education, self-support, and (actually helpful) social exchange on mental disorders on platforms such as TikTok. It is crucial, though, that professional producers of mental health content on TikTok are familiar with how to create videos that lead to engagement with their content on this specific platform to not fall short of its potential (McCashin & Murphy, 2023), even more so as there seems to be interest in professional mental health information (Karasavva et al., 2025) by the target audience.

### Limitations

Up to this point, there is no widely accepted procedure to evaluate short videos on social media. We tried to increase comparability by combining previously created ratings (Aragon-Guevara et al., 2025; Yeung et al., 2022) and standardized measures. Still, we think that a time-efficient measurement of reliability is crucial to evaluating and further promoting scientifically valid information online. While our study evaluated a total of almost 180 videos, one might argue that 30 videos per mental disorder will naturally only cover a fraction of what is to be found online.

As to the selection of diagnoses, it could be argued that the narcissistic personality disorder is not as clearly defined as other disorders since it is not officially classified in the ICD, for example. “Narcissism” is, however, one of the most popular mental-health hashtags on German-speaking TikTok. Since it is safe to assume that a layperson browsing TikTok would not know whether they are dealing with an official diagnosis or not and the aim in this study was to reflect the experience of the average TikTok user as closely as possible, the term was included nevertheless.

Finally, even though our study expands previous knowledge by analyzing the German-speaking TikTok, this might not be representative of other languages and cultures. Thus, our results should be replicated in other languages and potentially with other disorders.

## Supplementary Materials

The Supplementary Materials contain the following items (for access, see Mross et al., 2025S):

*TikTok Study Rating Guideline*: the rating guideline used by the two raters during the process of the study; including the specifically rating of correctness, mDISCERN and GQS.

MrossA. L.
TakahashiH.
KoelkebeckK.
LangenbachB. P.
 (2025S). Supplementary materials to "Insufficient quality of mental health information on German-speaking TikTok: A content analysis"
[Rating guideline]. PsychOpen. 10.23668/psycharchives.21394
PMC1295530841783472

## Data Availability

The videos and data sets generated or analyzed during this study are not publicly available but are made available for researchers upon reasonable request to the corresponding author. The rating manual is available as supplementary material.

## Preregistration

The study was not preregistered.

## Reporting Guidelines

The article follows the APA JARS-Quant reporting guidelines.

## References

[sp1_r1] Mross A. L. Takahashi H. Koelkebeck K. Langenbach B. P. (2025 S). Supplementary materials to "Insufficient quality of mental health information on German-speaking TikTok: A content analysis" [Rating guideline]. PsychOpen. 10.23668/psycharchives.21394 PMC12955308 41783472

